# Effectiveness of rehabilitation training combined acupuncture for the treatment of neurogenic bladder secondary to spinal cord injury

**DOI:** 10.1097/MD.0000000000017322

**Published:** 2019-09-27

**Authors:** Gui-fen Yang, Di Sun, Xin-hua Wang, Li Chong, Fang Luo, Cheng-bing Fang

**Affiliations:** a Department of Rehabilitation, Tongde Hospital of Zhejiang Province; b Department of Acupuncture, Moxibustion and Tuina, Xinhua Hospital of Zhejiang Province, Hangzhou, China.

**Keywords:** acupuncture, effectiveness, neurogenic bladder, rehabilitation training, safety, spinal cord injury

## Abstract

**Background:**

This study will aim to assess the effectiveness of the rehabilitation training (RT) combined acupuncture for the treatment of patients with neurogenic bladder (NB) secondary to the spinal cord injury (SCI).

**Methods:**

We will conduct a comprehensive literature search from the following databases from the inceptions to the present with no language limitation: PUBMED, EMBASE, Cochrane Library, SinoMed, Web of Science, Allied and Complementary Medicine Database, VIP, WANGFANG, Chinese Biomedical Literature Database, and China National Knowledge Infrastructure. Additionally, we will also search gray literature, including dissertations and conference proceedings. RevMan V.5.3 software will be used for the study selection, assessment of bias of bias, and data synthesis.

**Results:**

This study will synthesize the available evidence of RT combined with acupuncture for NB secondary to SCI, including episodes of urinary incontinence, urinary retention, urinary tract infection, bladder overactivity, quality of life, and adverse events.

**Conclusion:**

This study will determine whether RT combined acupuncture is an effective and safety therapy for NB secondary to SCI.

**Systematic review registration:**

PROSPERO CRD42019146127.

## 1 Introduction

Neurogenic bladder (NB) is an inconvenience disorder secondary to the spinal cord injury (SCI).^[1–3]^ Such condition comprises of urinary incontinence, urinary retention, and other lower urinary tract symptoms.^[4–6]^ It is reported that NB often negatively affects functional recovery, quality of life, and costs of health care and treatments.^[7–10]^ It has been estimated that about 80% SCI patients experience some levels of bladder dysfunction within 1 year postinjury.^[8,11,12,13]^ In addition, about 42% hospitalized SCI patients have urinary problems every year.^[8,11,12,13]^ Thus, it is very important to manage urological problems for both inpatient and community-based patients with SCI.^[14]^ These managements often consist of catheterization approaches, medication, surgery, electrical stimulation, Chinese herbal medicine, moxibustion, acupuncture, and rehabilitation training (RT).^[15–21]^ However, there is still insufficient efficacy for such single intervention. Fortunately, previous studies have reported that patients with NB secondary to SCI can benefit from the treatment of acupuncture combined RT.^[20,22,23]^ This study will assess the effectiveness and safety of acupuncture and RT for patients with NB secondary to SCI.

## 2 Methods

### 2.1 Ethics and dissemination

Ethical approval is not necessary because this study will not analyze specific patient data. The results of this study are expected to be published at peer-reviewed journals.

### 2.2 Eligibility criteria for study selection

#### 2.2.1 Type of studies

All randomized controlled trials (RCTs) will be considered for inclusion without limitations on language and publication status. Non-RCTs and uncontrolled clinical studies will be excluded.

#### 2.2.2 Type of participants

The patients diagnosed with NB in patients with SCI will be included without limitation of country, gender, and age.

#### 2.2.3 Type of interventions

For the experimental group, RT combined acupuncture therapy will be considered for inclusion. Any other interventions or combined with RT or acupuncture will be excluded.

For the control group, any treatments can be received for patients, except any forms of RT, acupuncture, or RT combined acupuncture.

#### 2.2.4 Type of outcome measurements

Primary outcomes include episodes of urinary incontinence and urinary retention. Secondary outcomes consist of urinary tract infection, bladder overactivity, quality of life, and adverse events.

### 2.3 Search methods for study identifications

We will comprehensively search electronic databases irrespective of language from the inceptions to the present: PUBMED, EMBASE, Cochrane Library, SinoMed, Web of Science, Allied and Complementary Medicine Database, VIP, WANGFANG, Chinese Biomedical Literature Database, and China National Knowledge Infrastructure. Detailed search strategy for PUBMED is presented in Table 1. Similar search strategies for other electronic databases will be adapted.

**Table 1:**
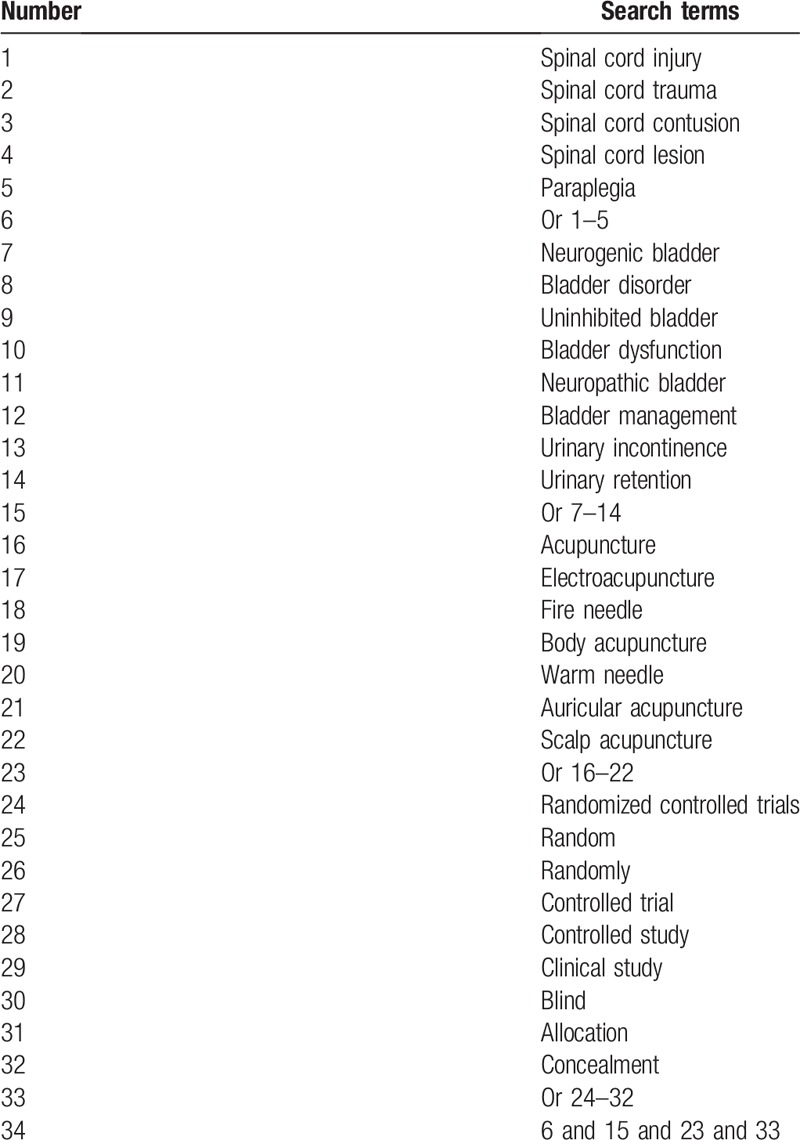
Search strategy for PUBMED.

Gray literatures will be implemented for further information including the dissertations, conference proceedings, and reference lists of relevant reviews.

### 2.4 Data collection and management

#### 2.4.1 Study selection

Strict eligibility criteria will be built before the study selection. Two authors will independently determine the eligibility of studies. Any duplicated and irrelevant studies will be excluded by scanning the titles and abstracts. We will read full text of remaining records if we are not sure whether the studies meet the eligible criteria. A 3rd author will help to resolve any disagreements between 2 authors. The screening process of study selection will be summarized in a flow diagram.

#### 2.4.2 Data extraction

A previous designed data extraction sheet will be developed, and study data will be collected by 2 authors independently. Any discrepancies between 2 authors will be judged by an independent arbitrator. The following information will be extracted from each eligible study, including general information (title, 1st author, publication time, etc), participants (age, gender, diagnostic criteria, sample size, inclusion and exclusion criteria, etc), methods (study design, study setting, randomization, blinding, concealment, etc), intervention details (types of interventions, comparators, dosage, frequency, duration, etc), outcomes (primary and secondary outcomes, safety, etc), and other information.

#### 2.4.3 Risk of bias assessment

Cochrane risk of bias tool will be utilized to evaluate the methodological quality of all eligible studies by 2 authors. Any different opinions between 2 authors will be solved by a 3rd author through discussion. Such tool will be assessed by 7 aspects and each item will be divided into 3 categories: high, unclear, and low risk of bias.

#### 2.4.4 Measures of treatment effect

For dichotomous outcome data, it will be calculated as relative risk and 95% confidence interval. For continuous outcome data, it will be calculated as the mean difference or standardized mean difference and 95% confidence interval.

#### 2.4.5 Assessment of heterogeneity

We will identify statistical heterogeneity among trial results using *I*^2^ statistic test. When *I*^2^ ≤50%, low heterogeneity is considered, while *I*^2^ >50%, significant heterogeneity is regarded.

### 2.5 Data synthesis and analysis

#### 2.5.1 Data synthesis

RevMan V.5.3 software is applied for statistical analysis. If two or more included trials are identified at the same outcome measurements, we will carry out meta-analysis. When *I*^2^ ≤50%, a fixed-effect model will be chosen, data will be pooled, and meta-analysis will be conducted if sufficient qualified studies will be included. When *I*^2^ >50%, we will apply a random-effect model for data pooling. We will also carry out subgroup analysis to explore possible causes. If there is still substantial heterogeneity after subgroup analysis, we will not pool the data, and we will report outcome results as a narrative synthesis instead of meta-analysis to summarize the characteristics and findings of the eligible studies.

#### 2.5.2 Subgroup analysis

We will perform subgroup analysis according to different interventions, controls, and outcome measurements.

#### 2.5.3 Sensitivity analysis

Sensitivity analysis will be conducted to identify whether the pooled results are robust by removing low quality studies.

#### 2.5.4 Reporting bias

We will conduct Funnel plots and Egger linear regression test if more than 10 RCTs are entered in this study.

## 3 Discussion

This study aims to analyze the effectiveness and safety of RT combined acupuncture on patients with NB secondary to SCI. Comprehensive databases will be searched from inceptions to the present. In addition, we will also search gray literature records to avoid missing any possible qualified studies. The results of this study will summarize the current evidence on the therapeutic effectiveness of RT and acupuncture for treating NB secondary to SCI, which may benefit clinical practice, patients, and policy makers.

## Acknowledgment

The authors thank Fuyang District Science and Technology Bureau Project (81403234) for the support. The funder did not have any roles in this study.

## Author contributions

**Conceptualization:** Di Sun, Xin-hua Wang, Li Chong, Fang Luo.

**Data curation:** Gui-fen Yang, Di Sun, Xin-hua Wang, Fang Luo, Cheng-bing Fang.

**Formal analysis:** Di Sun, Li Chong, Fang Luo, Cheng-bing Fang.

**Investigation:** Di Sun.

**Methodology:** Gui-fen Yang, Xin-hua Wang, Li Chong, Fang Luo, Cheng-bing Fang.

**Project administration:** Di Sun.

**Resources:** Gui-fen Yang, Xin-hua Wang, Li Chong, Fang Luo, Cheng-bing Fang.

**Software:** Gui-fen Yang, Xin-hua Wang, Li Chong, Fang Luo, Cheng-bing Fang.

**Supervision:** Di Sun.

**Validation:** Gui-fen Yang, Di Sun, Fang Luo.

**Visualization:** Gui-fen Yang, Di Sun, Xin-hua Wang, Li Chong.

**Writing – original draft:** Gui-fen Yang, Di Sun, Xin-hua Wang, Li Chong, Fang Luo, Cheng-bing Fang.

**Writing – review & editing:** Gui-fen Yang, Di Sun, Xin-hua Wang, Li Chong, Cheng-bing Fang.
